# The complete chloroplast genome of the Chinese Abelia *Lineae chinensis* (R.Br.) A.Braun & Vatke (Caprifoliaceae)

**DOI:** 10.1080/23802359.2018.1508386

**Published:** 2018-10-26

**Authors:** Jin-Liang Liu, Ying-Xi Qian, Cong Zhang, Ya-Qian Li, Yong-Hui Jin, Xue Wu, Meng-Di Li, Jun-Jie Wu, Xuan Zhou, Chao Shen, Rui-Hong Wang, Zhe-Chen Qi

**Affiliations:** a College of Life Sciences, Zhejiang Sci-Tech University, Hangzhou, China;; b Zhejiang Province Key Laboratory of Plant Secondary Metabolism and Regulation, Hangzhou, China

**Keywords:** *Lineae chinensis*, chloroplast genome, phylogeny

## Abstract

*Linnaea chinensis* (Caprifoliaceae), which inhabits in China and Japan, is commonly cultivated as an ornamental shrub. The complete chloroplast genome of *L. chinensis* was newly assembled in this study based on Illumina pair-end sequencing data. The full length of *L. chinensis* plastome is 155,813 bp. In total, 124 genes were identified, including 75 protein-coding genes, 8 rRNA genes, and 42 tRNA genes. The overall GC content of this genome was 38.4%. A further phylogenomic analysis including16 species from Adoxaceae and Caprifoliaceae was constructed.

*Linnaea chinensis* A.Braun & Vatke is a compact deciduous shrub with funnel-shaped, white flowers. It is distributed in China and Japan, and is a horticulturally important species in eastern Asia. *Linnaea chinensis* is one of the parental species of the garden hybrid *Linnaea* × *grandiflora*. This species was recently transferred from genus *Abelia* based on molecular evidence (Christenhusz 2013). No chloroplast genome information was reported so far for this genus. In this article, we assembled and characterized the complete chloroplast genome of *L. chinensis*. It will provide essential genetic data for molecular breeding and phylogenomic analysis in this genus.

The sample was collected from Chun’an, Zhejiang, China (Voucher No. ZSTU00877, deposited at Zhejiang Sci-Tech University). Total DNA was extracted from fresh leaves of *L. chinensis* individual using DNA Plantzol Reagent (Invitrogen, Carlsbad, CA, USA). The plastome sequences were generated using Illumina HiSeq 2500 platform (Illumina Inc., San Diego, CA, USA). In total, *ca*. 30.8 million high-quality clean reads (150 bp PE read length) were generated with adaptors trimmed. Then, CLC Genomics Workbench (CLC Bio, Aarhus, Denmark) was used for trimming, *de novo* assembly, and mapping to *Kolkwitzia amabilis* (KT966716) as reference. BLAST, GeSeq (Tillich et al. 2017), tRNAscan-SE v1.3.1 (Schattner et al. 2005), and GENEIOUS v 11.0.5 (Biomatters Ltd, Auckland, New Zealand) were used to align, assemble, and annotate the plastome.

The full length of *L. chinensis* chloroplast genome (GenBank Accession No. MH553932) was 155,813 bp. It comprises large single-copy region (LSC with 89,954 bp), a small single-copy region (SSC with 18,761 bp), and two inverted repeat regions (IR with 46,906 bp). The overall GC content of the *L. chinensis* cp genome was 38.4%. A total of 124 genes were contained in the cp genome (75 protein-coding genes, 8 rRNA genes, and 42 tRNA genes). Two genes of tRNA (*trnH-GUG*, *trnG-UCC*) had four copies and one of tRNA (*trnR-ACG*) has three copies. Ten genes had two copies, which included 1 PCG genes (*ndhB*), 5 tRNA genes (*trnL-CAA*, *trnV-GAC*, *trnI-GAU*, *trnA-UGC*, *trnN-GUU*), and all 4 rRNA species (*rrn4.5*, *rrn5*, *rrn16*, and *rrn23*). Among the protein-coding genes, two genes (*rps18* and *ycf3*) contained two introns, and other six genes (*atpF*, *ndhA*, *ndhB*, *rpl2*, *rpoC1*, *rps16*) had one intron each.

Using MAFFT v7.3 (Katoh and Standley 2013), we aligned 16 chloroplast genomes of species from Adoxaceae and Caprifoliaceae. A phylogenetic tree was drawn by statistical method of the maximum likelihood (ML) inference using GTR model with 1000 bootstrap replicates with RAxML v.8.2.1 (Stamatakis 2014) on the CIPRES cluster service (Miller et al. 2010). The result revealed that *L. chinensis* is sister to *Dipelta floribunda* and *Kolkwitzia amabilis* in Caprifoliaceae (Figure 1). We hope the newly characterized complete chloroplast genome of *L. chinensis* will provide essential genetic resource and background data for further phylogenetic study and breeding practices of the genus *Linnaea*.

**Figure 1.**
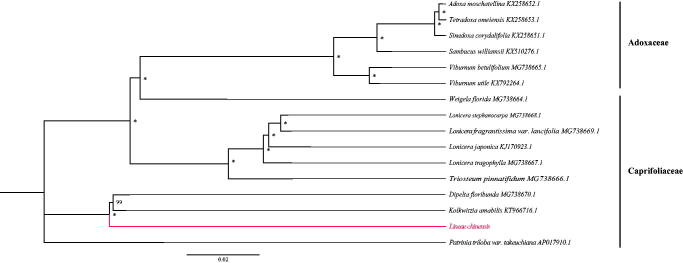
RAxML phylogeny of *Linnaea* based on 16 complete cp genomes (accession numbers were listed behind their names, and labels showed beside branches represent of bootstrap values, and “*” indicates a 100% bootstrap value).
